# Patterns and Drivers of Rodent Abundance across a South African Multi-Use Landscape

**DOI:** 10.3390/ani11092618

**Published:** 2021-09-07

**Authors:** Beatriz C. Afonso, Lourens H. Swanepoel, Beatriz P. Rosa, Tiago A. Marques, Luís M. Rosalino, Margarida Santos-Reis, Gonçalo Curveira-Santos

**Affiliations:** 1 cE3c—Centre for Ecology, Evolution and Environmental Changes, Faculdade de Ciências da Universidade de Lisboa, Campo Grande, 1749-016 Lisboa, Portugal; beatrizrprosa@hotmail.com (B.P.R.); lmrosalino@fc.ul.pt (L.M.R.); mmreis@fc.ul.pt (M.S.-R.); gcurveirasantos@gmail.com (G.C.-S.); 2 Department of Zoology, School of Mathematical & Natural Sciences, University of Venda, Thohoyandou 0950, Limpopo, South Africa; lourens.swanepoel@univen.ac.za; 3 Centre for Research into Ecological and Environmental Modelling, The Observatory, University of St Andrews, St Andrews KY16 9LZ, UK; tiago.marques@st-andrews.ac.uk; 4 Centro de Estatística e Aplicações, Departamento de Biologia Animal, Faculdade de Ciências, Universidade de Lisboa, 1749-016 Lisboa, Portugal

**Keywords:** non-invasive sampling, ecological modelling, management options, conservation

## Abstract

**Simple Summary:**

Wildlife ecological patterns are driven not only by environmental and biological contexts, but also by landscape-management schemes that shape those contexts. The present study aims to determine the effect of different environmental factors (including management schemes) on the occurrence patterns of a southern African small mammal community. Based on a landscape where three land-use contexts that differ in their levels of human presence and/or where activities coexist (private ecotourism reserve, mixed farms and traditional communal areas), and by using a body-size-based approach (i.e., using two size-based rodent groups—medium and small—as models), we found that the mean relative abundance of medium-sized species did not differ across the management contexts, but small species’ mean relative abundance was higher in the game reserve. The overall variation in rodent abundance was negatively affected by ungulate presence (possibly linked to a decrease in food availability) and by human presence (increased disturbance). Rodent abundance seems to be influenced by environmental gradients that are directly linked to varying management priorities across land uses, meaning that these communities might not benefit uniformly by the increased amount of habitat promoted by the commercial wildlife industry.

**Abstract:**

South Africa’s decentralized approach to conservation entails that wildlife outside formally protected areas inhabit complex multi-use landscapes, where private wildlife business (ecotourism and/or hunting) co-exist in a human-dominated landscape matrix. Under decentralized conservation, wildlife is perceived to benefit from increased amount of available habitat, however it is crucial to understand how distinct management priorities and associated landscape modifications impact noncharismatic taxa, such as small mammals. We conducted extensive ink-tracking-tunnel surveys to estimate heterogeneity in rodent distribution and investigate the effect of different environmental factors on abundance patterns of two size-based rodent groups (small- and medium-sized species), across three adjacent management contexts in NE KwaZulu-Natal, South Africa: a private ecotourism game reserve, mixed farms and traditional communal areas (consisting of small clusters of houses interspersed with grazing areas and seminatural vegetation). Our hypotheses were formulated regarding the (1) area typology, (2) vegetation structure, (3) ungulate pressure and (4) human disturbance. Using a boosted-regression-tree approach, we found considerable differences between rodent groups’ abundance and distribution, and the underlying environmental factors. The mean relative abundance of medium-sized species did not differ across the three management contexts, but small species mean relative abundance was higher in the game reserves, confirming an influence of the area typology on their abundance. Variation in rodent relative abundance was negatively correlated with human disturbance and ungulate presence. Rodent abundance seems to be influenced by environmental gradients that are directly linked to varying management priorities across land uses, meaning that these communities might not benefit uniformly by the increased amount of habitat promoted by the commercial wildlife industry.

## 1. Introduction

In South Africa, agricultural intensification, and overgrazing have led to profound land use changes [1]. Historically, most landscapes were converted into livestock farms and farmlands, either as intensive, extensive, or communally managed areas [2], leading to the destruction, degradation and/or fragmentation of natural ecosystems [3]. Consequently, such habitat destruction led to declines in wildlife populations and distribution in much of South African nonprotected areas [4]. 

However, the establishment of national policies attributing custodial rights over wildlife to landowners, prompted a transition in the governance of natural resources from the state to privates [5]. This political option led to widespread conversion of rangelands, i.e., farmlands and livestock farms, into areas dedicated to commercial wildlife industries, such as game ranching and private game/ecotourism reserves [6]. The positive conservation outcomes of these policies for economically valuable and charismatic species [7] is believed to have an umbrella effect on other taxa, mainly through the increased coverage, representativeness and connectivity of protected/restored habitats [6,8]. However, the effect of such management approaches is unexplored for most overlooked—but functionally important—taxa, such as rodents [9]. Thus, information on the ecological responses of less-charismatic taxa is needed to better gauge the complementary conservation role of South Africa’s private land.

In South Africa, game farms and private game reserves often coincide across relatively small scales, rooted in human-dominated landscapes (e.g., communal lands) [10]. These land uses have contrasting management priorities and, consequently, distinct impacts on the landscape structure and wildlife ecological patterns. In game farms, the main objective is to maximize the production of ungulates for meat or hunting, while in private game reserves the goal is to maintain charismatic species, promoting ecotourism-based activities [11]. Often, these wildlife-oriented land uses are surrounded by human-dominated areas with high levels of anthropogenic disturbance. The regional co-existence of all these land uses generates complex multi-tenured landscapes, usually divided by semi-permeable wildlife fences, influencing the biodiversity supported by each of these land uses [12].

Management actions directed to charismatic or valuable species may have cascading effects on rodents, usually overlooked and handled like pests [13,14,15]. However, it is crucial to understand the effect of human-induced land-use changes on rodent spatial patterns, as well as the underlying ecological mechanisms thereof, since rodents are fundamental for some ecosystem functions [16]. Rodents are primary consumers [16] and support a large community of predators [17,18], which makes them a vital link in food-chain structuring [19]. Moreover, they are considered useful indicators of ecosystem functioning as they are valuable tools to the description and monitoring of habitat integrity. For these reasons, rodents have been used as model species to understand how land use changes affects wildlife [16]. 

Several factors have been identified as influential in shaping rodent community and population structures, many of which are often determined by the landscape management options [20]. Some studies have indicated that vegetation type and structure are fundamental drivers of rodent occurrence and abundance [21,22,23]. For example, areas with greater herbaceous coverage favor rodents by providing shelter against predators, food, and adequate microclimatic conditions [24]. Studies have shown negative effects of overgrazing on small mammals’ abundance, by reducing the herbaceous stratum, increasing trampling risk and feeding competition with ungulates [9,24,25,26,27]. Regarding rodent distribution, it tends to be uniform when the habitat is favorable and resources are abundant. However, when disturbances increase the level of habitat heterogeneity, causing landscape fragmentation, their distribution is mostly clumped [28,29]. 

Rodents are not a homogeneous group, since different species may establish distinct relationships with the environmental and biotic components of the ecosystem. For example, larger rodents’ range over larger spatial scales than smaller rodents [30] and, therefore, are more susceptible to changes at this landscape level [31].

Changes in management priorities across South African multi-tenured landscapes will have a direct impact on these environmental drivers and, ultimately, in the distribution and abundance of small mammal species across and within management contexts. For instance, when management measures promote the abundance of ungulates (e.g., as prey for large carnivore populations in ecotourism reserves, or as hunting assets in game farms), grazing pressure will increase, negatively influencing the herbaceous strata [26]. Alongside with long dry and hot seasons [32], these conditions may lead to shrub encroachment, known to reduce food availability (leaves, seeds, and arthropods) for ground dwelling rodents [33]. Nevertheless, some rodent species are usually considered efficient colonizers of human shaped environments [13,34], as they are able to use human-related food resources due to their omnivore character [35].

Although the processes that regulate small mammals’ spatial distribution are known for some landscapes (e.g., woodland [29] and mixed forest [36]), there is a lack of information regarding the drivers of rodent-abundance patterns in African savannas (but see [9,37]), as well as how these vary across different management schemes. Here, we evaluated the variation in rodent abundance across three adjacent management contexts, spanning a private ecotourism game reserve, mixed farms and communally owned land, managed by Zulu tribal authorities [12], under the following two main objectives: (1) to estimate heterogeneity in small-mammal-abundance distribution (mean abundance and patchiness) across management contexts (game reserve, mixed farms and communal lands); and (2) to determine the main, fine-scale environmental factors affecting small-mammal-abundance patterns across land-use types. These objectives were tested in two size-based rodent groups, for a more detailed assessment of ecological responses.

Linked to these two goals, we tested four hypothetical drivers of rodent communities:

(i) An area-typology hypothesis, i.e., cumulative effect of management-induced changes to vegetation, grazing pressure, etc., creates area-specific differences in rodent abundance. Patchiness will also be tested to acknowledge in which area each group is more or less clumped, regarding their abundance values. Although the exact effect of area on rodent abundance is not fully predictable [37] (given the disturbance gradient) we expected the communal lands to have the lowest values of abundance and highest patchiness (i.e., more clumped), followed by mixed farms and the game reserve, with higher abundances and lower patchiness;
(ii) A vegetation-structure hypothesis, i.e., areas with higher herbaceous cover will have a positive influence on both rodent size-based groups, since it shapes the ability of the landscape to provide protection against potential predators [21,22,23,25,27,38];
(iii) An ungulate-pressure hypothesis, i.e., rodent species abundance is negatively influenced by the abundance of ungulates, since higher grazing pressure tends to decrease herbaceous land cover, increase disturbance due to the trampling effect, and increase landscape fragmentation [9,24];
(iv) A human-disturbance hypothesis, i.e., rodent species’ distribution is negatively influenced by human disturbance factors, such as the presence of domestic animals and households that may constrain species’ presence [14,39].

## 2. Materials and Methods

### 2.1. Study Area

**Figure 1:**
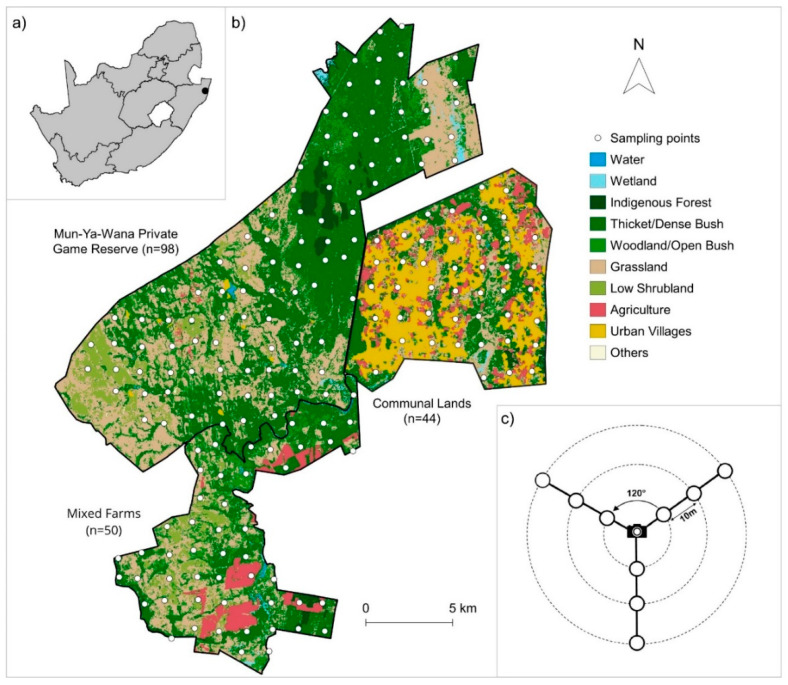
Location of the study area in South Africa, with the black dot representing the location of the study area in the Maputaland region of northern KwaZulu-Natal (**a**); landscape composition of the three studied areas with distinct management schemes–Mun-ya-wana private game reserve, mixed farms and communal land (Zulu tribal land)–with the location of the sampling points and the number of sampling points per area (in parenthesis) (**b**); each sampling point included a camera trap in the center and nine ink tunnels, distributed in a Y shape (open circles represent ink tunnels) (**c**).

This study was implemented in the Maputaland–Pondoland–Albany Biodiversity Hotspot [40] in northern KwaZulu-Natal, South Africa. Our specific study area is characterized by a spatial gradient of human intervention, ranging from the Mun-ya-wana private game reserve (less subject to human associated activities), to mixed game farms and to communally managed lands, where two distinct Zulu communities are settled (Figure 1b). The Mun-ya-wana private game reserve (27°40′ S–27°55′ S; 31°12′ E–32°26′ E) represents the union of several properties without internal fences, managed by private owners whose goal is to explore eco-touristic products, therefore promoting wildlife and habitat conservation. Those management objectives are commonly related with a more sustainable use of wildlife, typically wildlife-viewing tourism [41]. The reserve is surrounded, to the South, by a mosaic of commercial game ranches for the production of wild ungulate species, occasionally mixed with domestic cattle [42] (hereafter mixed farms) and represents large expanses of natural habitat with low human density. Communal lands to the east are composed of households, interspersed with pasture areas and semi-natural vegetation. The region is characterized by a warm-temperature climate, with a humid and hot summer (October to April), according to the Köppen–Geiger classification. Mean monthly temperatures range from 19 °C in July to 31 °C in January, and the average annual precipitation is 800 mm [43,44]. Elevation ranges from 3 m to 304 m above sea level [45], dominated by a similar mixture of vegetation throughout the area (bushveld, woodland and grassland) [46] (Figure 1b). Nevertheless, the game reserve hosts a higher diversity and abundance of pristine habitats, such as indigenous forests, while mixed farms are mainly composed of pasture areas (low shrubland and grassland–Figure 1). Contrarily, communal lands have the lowest proportion of vegetation and the highest cover of urban–village occupation (Figure 1).

### 2.2. Rodent Sampling

Rodents were sampled between October and November 2017 (the southern hemisphere’s spring) using ink-tracking tunnels [42], left active in the field for four consecutive nights (open circles in Figure 1c). Ink-tracking tunnels were made of robust corrugated plastic (55 × 10 × 10 cm), open on both ends to allow rodents to enter. Both entrances of the tunnel are equipped with an adhesive paper with the glue side up, and an ink pad (12 × 10 cm) was placed in the floor center [47] (Figure S1B). In the middle of the tunnel, a small PVC-pipe section, hanging from the ceiling, was installed, and contained bait composed of a mixture of peanut butter, oatmeal and sunflower oil [46]. The pipe was used to prevent the consumption of the bait by the animals entering/crossing the tunnel. The ink tunnels were placed on the ground, grouped in clusters of nine, in a Y formation, 10 m apart from each other (Figure 1c). The arms of the Y formation were 120 degrees apart (Figure 1c). This design provided an adequate spatial coverage in relation to the home-ranges of the rodent species, also ensuring some level of independence between sampling units, considering the mean distance between sites (see below). After the four-day sampling period, the plates of each ink tunnel (containing footprints and tracks) were photographed individually, always at the same distance and with a reference scale.

The footprint data was used to estimate rodent relative abundance, using the proportion of the tunnels with records (track index; TI–for more details see Supplementary Materials) [48]. To ensure that this approach captured spatial heterogeneity in relative abundance, we conducted a small trial, comparing the abundance indices derived from ink tunnels to those obtained from live-trapping (see Supplementary Materials, PART A). As track identification at the species level is very time consuming and not viable in large-scale studies, and as distinguishing footprints from similar-sized species is very difficult and bias prone, we opted for dividing tracks into groups based on track size (for more details see Supplementary Materials PART A; Figures S1A and S2A, Table S1A). Rodent footprints were grouped into three different size-based groups per body length/weight, assuming a relation between rodent body length/weight and footprint sizes [49,50]: small (body length: 50–100 mm), medium (100–150 mm) and large rodents (150–200 mm) (Figure S2B). Sampling intentionally took place outside the breeding season (which peaks in the wet season, [51]), in order to avoid grouping juveniles in the wrong size-based group. However, considering the low number of detections of large rodents in ink-tracking tunnels, we only analyzed the data from small- and medium-sized rodents (see Results). The most common species captured during live trapping and linked to each group were *Mus minutoides* and *Dendromus melanotis* for small rodents, *Mastomys natalensis* and *Saccostomus campestris* for medium rodents and *Otomys angoniensis* and *Rattus rattus* for large rodents (Table S2A). 

### 2.3. Environmental Variables

Vegetation structure variables were collected using two different approaches: field measures and remote-sensed products [52]. All variables collected have been previously detected as influential to rodent presence elsewhere (e.g., vegetation cover) [21,23]. Shrub-and-grass cover were visually estimated and assigned the corresponding Edwards classification category [53] (see Table 1 for details), within a 30 m radius buffer, centered on the ink tunnel’s Y formation. Regarding the land use, the predominant categories were selected (thicket, grassland, sand forest and urban villages) and, for each buffer, was assigned the category with the highest cover. According to the type of crops present in the study area, the harvesting season occurs mostly between April and June [54], not coinciding with the study period. Therefore, we assumed that there would be no influence of crop productivity on the distribution/abundance of rodents in our study. The percentage of tree cover was assessed based on the Global Forest Watch database (Table 1). We also selected the Normalized Difference Vegetation Index (NDVI), widely used as a vegetation productivity proxy, collected from Landsat 8 Images [55].

Variables of ungulate pressure and human disturbance were collected from Curveira-Santos et al. [12] camera-trap surveys. Cameras, located in the center of the Y formation, were active for 60–90 days, and attached to a tree or metal stake, 30 cm above the ground, without any bait and set to photograph at minimum delay (1 s for daytime and 30 s for night-time) (see [12] for details). Each of the defined ink-tunnel clusters (i.e., one cluster includes nine ink tunnels and one camera-trap; Figure 1c) were spaced approximately 1.4 km apart (Figure 1b). In total, were sampled 196 points: 100 points in Mun-ya-wana eco-tourism/game reserve, 50 points in mixed farms and 46 points in communal lands. Capture rates, expressed as the number of independent camera records (>1 h interval between photographs of the same species, per 100 trap-days) for livestock (cows and goats), wild ungulates and human disturbance, were used as surrogates of disturbance in the modeling procedure (Table 1). 

Wild ungulates were grouped according to two criteria: weight, since trampling is one of the main negative impacts of ungulates on rodents [26], and/or the fact that they are actively managed in all studied areas (Table S1B). Only ungulates weighing between 45–200 kg and actively managed were used in the analysis, since they are more abundant than other ungulates, as they are present throughout the areas under study, and because they have a greater impact on rodents, due to their weight (Table S1B). Livestock were also separated in two weight classes: i.e., goats and cows. 

### 2.4. Data Analyses/Modelling

#### 2.4.1. Spatial Patterns of Rodent Relative Abundance Across Areas and Size-Based Groups

Differences in mean abundance values of size-based groups (small and medium) between study areas (Mun-ya-wana game reserve, mixed farms and communal lands) were tested using GLM with 3-level area covariate and binomial error distribution. The magnitude of patchiness in each area was ascertained by spatial-point pattern analysis of count data using Lloyd’s index of patchiness [62]. A Lloyd’s index of 1 indicates a random distribution, whilst one <1 suggests uniformity and >1 patchiness.

#### 2.4.2. Influence of Environmental Variables on Rodent Relative Abundance

Due to the high number of candidate variables and to avoid multicollinearity bias, we first estimated the nonparametric Spearman’s correlation (*r_s_*) using the “psych” R package [63]. When a high correlation between two covariates was detected (*r_s_* ≥ 0.7; [62]), the variable that was less correlated with the dependent variable was excluded from the analysis [64]. 

The influence of all candidate variables on rodent relative abundance was tested using a boosted-regression-tree (BRT) approach, implemented with the “gbm” package [65] in R [66,67]. This modelling technique encompasses the advantages of regression trees (e.g., predictor variables can be of any type, analysis is insensitive to outliers and can accommodate missing data [68]), overcoming their low predictive capacity through the boosting algorithm [69]. The final model is a linear addition of several regression models in which the simplest term is a tree [68,70].

Boosted-regression-tree models are resilient to model overfitting but, to have a better predictive performance, we defined, a priori, the model’s input parameters based on Carslaw and Taylor’s suggestions [70]. In BRT, learning rate (lr) is the shrinkage parameter that controls the contribution of each tree to the model, and tree complexity (tc) determines the number of nodes in a tree and, consequently, its size. These two parameters control the number of trees in the model, while the bag fraction (0.5) selects the proportion of data being used at each step [61,70,71]. All models were fitted to allow interactions using a ten-fold cross validation to determine the optimal number of trees for each model. The largest learning rate and the smallest tree complexity were selected to allow a minimum of 1000 trees in the BRT fitting process (see [68]). Non-informative variables were removed during the fitting process, allowing the simplification of the set of variables [68]. This simplification consisted of defining how many variables the function can test to remove, based on relative influence and total number of variables. Then, a graph was produced showing differences in the predicted deviance according to several scenarios, each one with a different number of variables removed. Next, the number of variables to eliminate was decided, and they were removed in order of minor relative influence. We defined a threshold value and only reported the interactions with relative influence values >10%. The final relative influence of each variable was calculated by averaging the number of times a covariate is used for splitting, weighted by the squared improvement to the model as the result of each split. It is then scaled, such that the values sum to 100 [72]. Fitted values were plotted in relation to the most important predictors, revealing their effects on rodent abundance. Explained deviance was calculated using the following formula from Abeare (2009) [73]

$$D^{2}=1-\left(\frac{residual\;deviance}{total\;deviance}\right)$$

The 95% confidence intervals of each variable were estimated for the fitted function by taking 500 bootstrap samples of the input data, with the same size as the original data. A BRT was fitted to each sample, and the 5th and 95th percentiles were calculated for the points of each function. Models were built separately for small- and medium-sized rodents. For each model performed, interactions between typology and the other influential independent variables (i.e., relative importance above >10%) were estimated, to evaluate context-dependency in the influence in the effect environmental variable associated with the management context. All analyses were implemented in R via R Studio Version 1.1.463 [66,67].

## 3. Results

### 3.1. Spatial Patterns of Rodent Abundance Across Areas and Size-Based Groups

**Figure 2:**
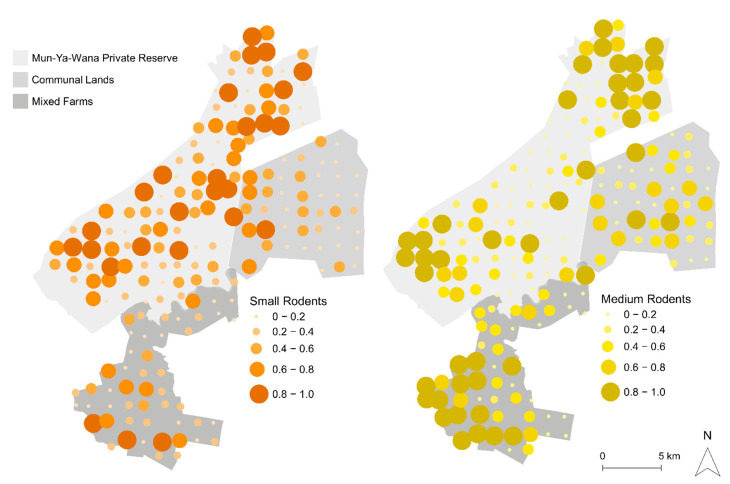
Map of the study area showing rodent distributions: small-size rodents are in orange and medium-size rodents in yellow. The size of each point is equivalent to abundance value, as indicated in the respective legend.

**Figure 3:**
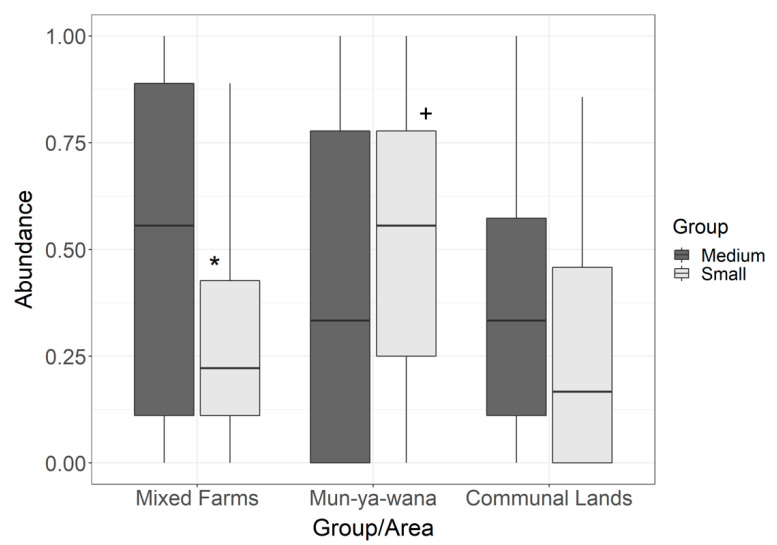
Boxplot of medium and small rodents’ relative abundance in the three management-type zones monitored: game (mixed) farms, Mun-ya-wana game reserve and communal lands. Based on the GLM test, * indicates a significant difference between size-based groups in mixed farms (*p* = 0.011), + indicates a significant difference between Mun-ya-wana game reserve and remaining areas for small rodents (*p* = 0.016).

From the 192 sampling points monitored, 85% presented small rodent tracks, while 76% detected the occurrence of medium rodents, with an overlap in 35% of sites and inter-area variation in detection (i.e., number of tunnels with signs/total number of tunnels, Table S2B). Mean abundance in Mun-ya-wana game reserve was 0.52 ± 0.26 (mean ± SD) for small rodents and 0.43 ± 0.34 for medium rodents; in mixed farms, 0.31 ± 0.21 for small rodents and 0.52 ± 0.32 for medium rodents; and in communal lands was 0.26 ± 0.23 for small rodents and 0.36 ± 0.24 for medium rodents (Figure 2). Regarding the GLM result for size-based groups, it revealed significant differences in relative abundances only for small rodents, between Mun-ya-wana game reserve and the remaining areas (Table S3B, Supplementary Materials). No significant differences were detected in relative abundances of medium rodents between areas (Figure 3). Between groups, significant differences were only found in mixed farms (Table S3B, Supplementary Materials), with medium rodents being more abundant (0.52 ± 0.37) than small-size rodents (0.31 ± 0.26) (Figure 3). Based on these results, the effect of environmental drivers on rodent abundance was evaluated separately for each of the size-based groups. 

#### Rodent Patchiness

Lloyd’s Index of Patchiness revealed that for every area and size-based group, all abundance values were aggregated (γ > 1; Table 2). Both medium and small rodents are heterogeneously distributed within the three study areas (Figure 3), demonstrating a heterogeneity gradient. According to Table 2, we can observe that the highest values for small rodents are in communal lands, followed by mixed farms and finally, the game reserve. For medium rodents, there is a greater clustering pattern in the game reserve, followed by communal lands and mixed farms. With these results, it is possible to state that the abundance patterns differ between the size-based groups, and within each area.

### 3.2. Drivers of Abundance

Capture rate of goats and cows were both correlated with human presence (*p* = 0.75; *p* = 0.76, respectively), and intercorrelated (*p* = 0.79). Therefore, both former variables were removed from the analysis. 

#### 3.2.1. Small-Size Rodents

**Figure 4:**
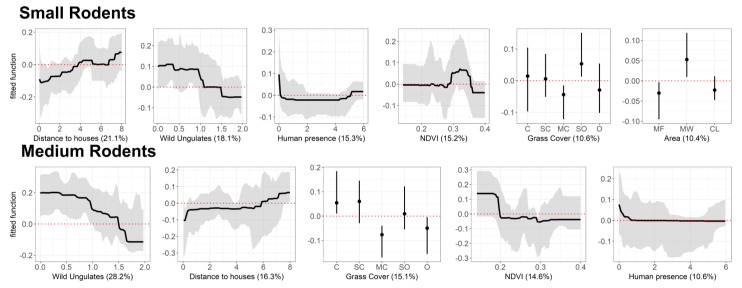
Variation in abundance (fitted function) predicted from the boosted-regression-tree (BRT) models, for the most important predictors of rodent abundance (relative importance > 10%). The 95% confidence intervals of each variable are represented in grey and the red dotted line represents the boundary between the positive and negative effects. Functions are continuous for all variables, except for grass cover and area–grass cover: C—continuous, SC—sub-continuous, MC—moderately closed, SO—semi-open, O—open; area: MF—Mixed farms, MW—Mun-ya-wana, CL—Communal lands. A common scale is used on the vertical axis for all plots (see Table 1 for variable units).

**Figure 5:**
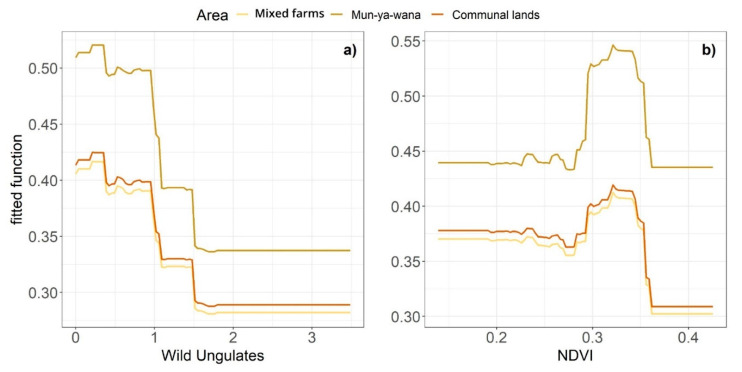
Interaction between area typology and (**a**) wild ungulates (**b**) NDVI. Each line represents the variation of small-rodent abundance in the respective area (see color legend).

The predictive deviance for the BRT model produced for small rodents was 38.8%. After the simplification of the model, and consequent removal of two variables, predictive deviance increased to 50%, indicating that the final model explained an important part of the total variability [68]. Distance to houses, wild ungulates, human presence, NDVI, grass cover and area were identified as the most influential drivers of small rodent abundance (Figure 4). Small rodents were more abundant in areas far from human settlements, with lower abundances of wild ungulates and low presence of humans. Regarding the NDVI, values between 0.29 and 0.35 affect positively the abundance of small rodents. Semi-open grass cover had the most positive effect on small rodent abundance, as well as the Mun-ya-wana ecotourism/game reserve. Interactions with area typology within this model were found for wild ungulates (0.20, interaction size) and NDVI (0.34). As it is possible to see, in the Figure 5, that the most evident and distinct responses for both variables occur in Mun-ya-wana game reserve, revealing a clear influence of this area on wild ungulates and NDVI.

#### 3.2.2. Medium-Size Rodents

For this rodent group, the initial predictive deviance of the model was 40.6%, but, after the removal of one variable during the model simplification, the predictive deviance increased to 50%. The set of variables identified as important for this group was very similar to that described for the previous rodent groups (Figure 4). Medium-size rodents’ abundance was also higher in areas with low abundance of wild ungulates, human presence and which were far from human settlements. However, this group seems to thrive in more continuous grass cover and it is positively affected by low values of NDVI (0–0.18). Contrarily to the small-rodents group, this model did not include the area variable, which may indicate a lower relevance of area typology in shaping the abundance patterns of these rodents. 

## 4. Discussion

Rodent abundance, although often an unheeded aspect of conservation management, is crucial to understand ecosystem functioning, since rodents are primary consumers [16] and support a large community of predators [17,18], making them a vital link in food-chain structuring [19]. In our study area, spatial heterogeneity in rodent-abundance patterns appears to be influenced by environmental gradients that are directly linked to varying management priorities across land uses (e.g., ungulate pressure associated with wild game), which means that these rodent communities, and groups within these communities, might not benefit uniformly from the increased amount of habitat promoted by the commercial wildlife industry.

### 4.1. Context-Specific Responses and Variation Across Management Schemes

Area typology was an important abundance driver for small rodents (thus, just partially supporting our first hypothesis-H1), with higher abundances being estimated for Mun-ya-wana game reserve than for the remaining areas. Medium-size rodents did not show any significant differences in their abundance between areas (Figure 3).

The difference in small rodent abundance between areas (Figure 3) is supported by the interactions of the NDVI and wild ungulates abundance with the area typology (Figure 5). Overall, small rodent abundance decreased with an increase in wild ungulate abundance, irrespective of the management scheme, as predicted (H3: ungulate pressure hypothesis). Similarly, small rodent abundance increased with an increase in NDVI. However, the game reserve displayed a higher small rodent abundance, relative to the other land uses, and there is a differential effect of wild ungulates and NDVI on abundance between areas. Within the game reserve, these variables have a greater influence on this group probably due to the applied management practices. The greater variation in small rodent abundance in response to variation in wild ungulate abundance in Mun-wa-wana game reserve may be driven by the greater vegetation spatial heterogeneity of this area. The game reserve has a greater habitat heterogeneity compared to the other study areas due to better conservation derived from its protection status. This habitat heterogeneity results in a heterogeneous distribution of wild ungulates, owing to differences in habitat preference or selection (e.g., [74]). Thus, this wider variation of ungulate abundance across the reserve induces a more pronounced response in rodents, leading to the detected typology effect. Regarding the NDVI, the response may be influenced by the same factor (better conservation status of native forests-sand forests), which assure a lower disturbance regime, and thus create conditions to support a more abundant rodent community. However, the conservation character of some environments may induce the opposite trend in other taxa. Studies that analyzed the influence of protected areas in the conservation of small mammals found that these areas exhibit lower abundances compared to neighboring areas, since their conservation aims is mostly focused on wild ungulates and predators [37]. This induces small mammals’ movement to nearby areas, such as farms and agriculture lands, where they can find more resources (e.g., food) [9], and sometimes lower predation pressure. A study conducted in the same studied game reserve, based on live trapping measures, revealed a higher abundance of small mammals in adjacent farms and former cattle farms [9]. This pattern seems to be corroborated by our study data, but only for medium rodents that are less abundant in the more protected area (i.e., Mun-ya-wana game reserve). Small rodents respond differently, and the pattern may be associated with the environmental conditions provided by the game reserve, that seem to promote this group abundance. As mentioned above, the game reserve has a greater habitat heterogeneity derived from its protection status. This allows the conservation of certain vegetation patches that do not thrive in the other two areas. In this case, the NDVI values that promote a higher abundance of small rodents (between 0.28–0.35, Figure 4) correspond to native forest that exist in greater coverage in the game reserve (i.e., sand forests, Figure 1). Despite a greater abundance of wild ungulates and possible predators, the presence of these native habitats establishes more favorable conditions for small rodents. Considering that these rodents use the landscape on a smaller scale due to their size [30], these minor patches of vegetation create a significant difference in the abundance of this group.

Rodent abundances vary not only between areas (linked to areas specificities, and small mammals’ requirements), but also show an inter-group variation within areas. The spatial variation of abundances within-areas seems to be linked to the type of management implemented in each area that affects the vegetation structure and thus may have important implication in species conservation [9]. Lloyd’s Index supports that aggregation levels differ between size-based groups, since rodents preferentially aggregate in different areas (medium-size rodents in mixed farms and small rodents in Mun-ya-wana game reserve), which supports an allopatric distribution of both rodent groups. Furthermore, the highest abundances of each size-based group occurred in distinct areas (small in Mun-ya-wana, medium in mixed farms). Places where rodents occur in a more regular pattern, usually have better conditions (i.e., higher, and more regularly distributed resources), while sites where rodent distribution is more aggregated/clustered, indicate a more heterogeneous distribution of resources [75]. Our results show that the lowest values of Lloyd’s Index i.e., less patchy distribution, match the highest abundance values for both groups. This pattern is verified for small rodents in the game reserve and medium rodents in mixed farms (Table 2). Area typology influences the patchiness, since conditions will be more or less suitable for rodents according to the type of management applied (e.g., reserve and communal lands; [76]). A greater patchiness may lead to isolated populations, causing more sensitive species to disappear [75]. Thus, it is crucial to determine which type of management best promotes rodent abundance.

### 4.2. Fine-Scale Environmental Drivers of Rodent Abundance Across the Landscape

Our data also reveals that the abundance of both rodents groups is overall promoted by grass cover, which supports our second hypothesis (H2). However, the type of grass cover that enhances rodent abundance varies between groups. While medium-size rodents reached higher densities in continuous grass cover, small rodents are more abundant in semi-open grass cover. Grass cover, especially continuous layers, can provide protection against potential predators [25,27], reducing predation risk, and therefore allowing medium-size rodents to reach higher abundances. The different results might be associated to habitat preferences. Small rodents occurred predominantly in forested savanna areas (ex. Mun-ya-wana game reserve center area), while medium rodents occurred predominantly in open savanna areas (ex. north and south areas of the game reserve–see Figure 1 and Figure 2). The continuous grass cover patches may be more important in these open areas, since they provide an efficient protection against predators [24]. In forested regions (where small-size rodents seem to be more abundant), grass cover may be less important compared to its potential cover under better conservation of native forests, which guarantees a greater diversity of microhabitats and assures a lower disturbance regime, thus creating conditions to support a more abundant rodent community. 

The presence of ungulates (wild or domestic) has been associated with a reduction of habitat quality for rodents, by decreasing the availability of food and shelter for these small mammals [26,37]. This general pattern is reflected in our results, corroborating our third hypothesis (H3), i.e., species abundance is negatively influenced by the abundance of ungulates. This negative impact of ungulates may be linked to their impact on vegetation [26], since higher grazing pressure tend to decrease herbaceous land cover [9,24]. A study conducted in central Kenya showed an increase in small mammals’ abundance in the absence of ungulates, revealing the existence of food competition between ungulates and African rodents [77]. Although being omnivores, rodents feed mainly on seeds and grasses [78], which are highly depleted when ungulates are present. Furthermore, the ungulates trampling impacts on small mammals are also a possible explanation for this negative influence, since the soil compaction due to ungulates movements hampers burrows maintenance [26,79]. Other studies highlighted the impact of a reduction of the herbaceous layer, as it decreases refuge availability and increases predation risk by improving small mammals’ detection by predators [31,80,81,82]. Therefore, these two-fold effects (decrease in food and shelter availability), acting in isolation or in synergy, may be the underlying processes that constrain rodent abundance in the presence of ungulates. 

The distance to human settlements and human presence are also two factors that we identified as having a negative effect on both rodent groups’ abundance, which corroborates our fourth hypothesis (H4). Rodents revealed lower abundances in areas closer to houses, especially in communal lands, the area with the highest density of settlements (while houses are almost absent from the other two areas). Thus, the effect of this variable cannot be linearly interpreted as a distance to the nearest house, but probably as a distance to the communal lands themselves, as both groups’ abundances are low in this area (see Figure 2). The average abundance values confirm that the least preferred zone for both groups of rodents are the communal lands, as it is the place where the lowest values of abundance were estimated (Figure 3). However, these negative effects of anthropic disturbance may also be linked to the presence of domestic animals (livestock and goats), that occur concomitantly with settlements, and that also negatively affect rodent abundance, due to the same processes described above for wild ungulates [26].

This different patterns between rodent groups, as well as the variation of the drivers and their importance on the abundance variation of both species, supports the division of our dataset into size-based groups. This means that not only rodents should be taken into consideration, but also heterogeneity within rodent communities, which is important given their different functional roles (e.g., as prey, as consumers–granivory and insectivory–and seed dispersers). 

Although we acknowledge some limitations of this approach, based on footprint size, we have tried to minimize this by sampling only in seasons where the misclassification effect of juveniles’ presence is negligible. Nevertheless, this time-limited sampling hampers the validity of extrapolating results. Interpretation of the overall (annual) pattern of abundances’ spatial distribution must be done with care. Rodents numerically respond to variations in rainfall and food availability, which vary throughout the year. Thus, by sampling in only one season, we may have gotten a partial image of the processes shaping rodent abundance. However, in terms of wildlife management and conservation, it is always better to have a partial understanding of the ecological patterns and processes than having none.

## 5. Conclusions

Our study contributes to the current view that landscape-management options shape the ecological patterns of species, by modifying the composition and structure of habitats. Moreover, responses to land composition are species/group-specific. These results highlight the need to expand conservation actions beyond protected areas. For biodiversity conservation to succeed in these habitat mosaics, landscape-level policies and management are required to integrate both protected and managed areas, as the later also host a large number of species, acting as a metapopulation source-sink. We encourage future work that evaluates the transferability of our findings to other southern African multi-use landscapes. 

## Figures and Tables

**Table 1:** Environmental variables used in the modeling procedure used to assess the determinants of rodent abundance, collected in the field, from camera-trapping or based on remote-sensing data (GIS-based variables). The variable description, acronym, range, resolution and source, as well the reference that support their influence on rodent presence/abundance, are listed. H1—Hypothesis 1; H2—Hypothesis 2; H3—Hypothesis 3, H4—Hypothesis 4.

Variable Acronym	Description	Mean/Range	Resolution	Source	Supporting References
AREA TYPE (H1)					
Area	Managment context	Mixed farms Mun-ya-wana Communal lands	Collected at point	-	[37]
VEGETATION STUCTURE (H2)					
Tree_Cover	% Tree Cover	30.80/6–72%	30 × 30 m	Global Forest Watch https://www.globalforestwatch.org/ (16 April 2019)	[27,56]
Shrub_Cover	% of Shrub cover	Continuous (C)—76–100% Semi-continuous (SC)—51–74% Moderated closed (MC)—26–50% Semi-open (SO)—11–25% Open (O)—0–10%	30 m buffer	Visually estimated	[22,27,39,57,58,59,60]
Grass_Cover	% of Grass cover	Continuous (C)—76–100% Semi-continuous (SC)—51–74% Moderated closed (MC)—26–50% Semi-open (SO)—11–25% Open (O)—0–10%	30 m buffer	Visually estimated	[22,25,38,57]
Land_use	Land use categories	Thicket Grassland Sand Forest Urban Villages	30 m buffer	2013–2014 National Land Cover South Africa-SASDI http://www.sasdi.net/ (16 April 2019)	[21,22,23]
NDVI	Normalized difference vegetation index calculated from Landsat images	0.48/0.28–0.67	30 × 30 m	Landsat 8 https://earthexplorer.usgs.gov/ (18 April 2019)	[60,61]
UNGULATE PRESSURE (H3)					
Goats	Capture rate of goats (number of records per 100 days of trapping)	0.16/0–1.88	Collected at point	Camera-trapping survey	[9,24,26]
Livestock	Capture rate of cows (number of records per 100 days of trapping)	0.20/0–3.17	Collected at point	Camera-trapping survey	
Wild Ungulates	Capture rate of ungulates (number of records per 100 days of trapping)	0.750/0–3.48	Collected at point	Camera-trapping survey	
DISTURBANCE VARIABLES (H4)					
HUMANS	Capture rate of humans	0.84/0–10	Collected at point	Camera-trapping survey	[39]
DIST	Distance to houses	2.738/0.031–9.867 km	Collected at point	Camera-trapping survey	

**Table 2:** Results of Lloyd’s Index of Patchiness per study area and rodent size-based group (small and medium) (γ).

Area	Lloyd’s Index of Patchiness (γ)	
	Small	Medium
Mun-ya-wana game reserve	1.128	1.529
Mixed farms	1.372	1.296
Communal lands	1.528	1.306

## Data Availability

The datasets generated during the current study are available from the corresponding author on reasonable request.

## Supplementary Materials

The following are available online at https://www.mdpi.com/article/10.3390/ani11092618/s1. PART A—Comparison of ink-tracking tunnels with live-trapping for track index validation. Figure S1A: example of forefeet tracks of the three functional groups; Figure S2A: track measures in mm from the three functional groups; Table S1A: Dunn’s test for the four track measurements between groups; Table S2A: list of species occurring or possibly occurring in the region. PART B—Figures and tables additional to the manuscript. Figure S1B: ink-tracking tunnel scheme; Figure S2B: scheme of the method used to measure the 100 random tracks; Table S1B: categories used to describe the abundance of wild ungulates detected during the camera-trapping campaigns; Table S2B: percentage of rodent detection in each area per functional group; Table S3B: linear regression models between size-based groups and areas. References [83,84,85,86,87,88,89] are cited in the Supplementary Materials.
