# Supplementary material for: Patterns and Drivers of Rodent Abundance across a South African Multi-Use Landscape
Source: Animals (Basel). 2021 Sep 7;11(9):2618. doi: 10.3390/ani11092618 (PMC8471473; doi:10.3390/ani11092618)
Supplement: Supplementary file 1 [file animals-11-02618-s001.zip › animals-1346729-supplementary.pdf]

animals

MDPI

Supplementary Materials:

PART A – Comparison of ink tracking tunnels with live-trapping for track index validation

Rodent sampling

The estimation of rodent abundance based on ink tunnels may be affected by some bias, as it does not provide information on the individuals of the population. Therefore, we implemented, simultaneously to the ink tunnel monitoring, a live-trapping survey, restricted a total of 18 sites (10 sites in Mun-ya-wana game reserve, 5 in mixed farms and 4 in communal lands), to test if the abundance estimations derived from ink tunnels were correlated to those obtained from live-trapping, thus validating the use of ink tunnels abundance estimations. In each sampling site we set a 7 × 7 grid of Sherman traps (H. B. Sherman Traps, Inc., Tallahassee, Florida), 10 meters apart from each other. Traps were baited with a mixture of peanut butter, oatmeal and sunflower oil, being active for 3–5 days and checked every morning, midday, and evening. Captured animals were handled to allow species identification, and animals divided into three groups, according to their size and weight (following the strategy used for ink tunnels data): small, medium and large sized rodents. The live-trapping data was used to estimate rodent relative abundance, using Pounds relative abundance index [83,84] for both size-based groups (see data analysis). For comparison purposes we only used the first two groups to match data collected in ink tunnels. All live-trapping procedures were performed according to ethical clearance approval (University of Venda - SMNS/17/Z00/04/0905).

Data analysis and modelling

To compare the effectiveness of ink-tracking tunnels versus live-trapping i.e., ability to capture small mammals' evidence, we estimated a track index (TI), based on the proportion of tunnels with small mammal tracks [49], for two different size-based groups, per site. We excluded large sized small mammals due to the limited number of detections. A Pearson correlation was then applied between track index and Pounds relative abundance index [83].

Results

3.1. Rodents' functional groups distinction and tracking index validation

Rodents' footprints were grouped into three different size-based groups, based on their body length and footprint size (Figure S1). The three groups have distinct means for both length and width on the forefeet and hindfeet (Figure S2). According to Shapiro-Wilk test applied to the measures, data is normally distributed in all cases ($p < 0.05$), with exception for medium rodents' forefoot length and small rodents' hindfoot length. Levene's homogeneity of variances test revealed that all variables showed heteroscedasticity ($p < 0.05$) [85]. By failing one of the ANOVA assumptions, the Kruskal-Wallis non-parametric test was applied [86], whose results showed a significant difference between the three groups for all measures and both feet ($\chi^2_{(2)} = 82.09$, $p < 0.001$) [87]. Dunn's test results showed significant differences between small rodents and the other functional groups ($p < 0.001$). Medium and large rodents showed differences between, although not as significant ($p < 0.05$) (Table S1).

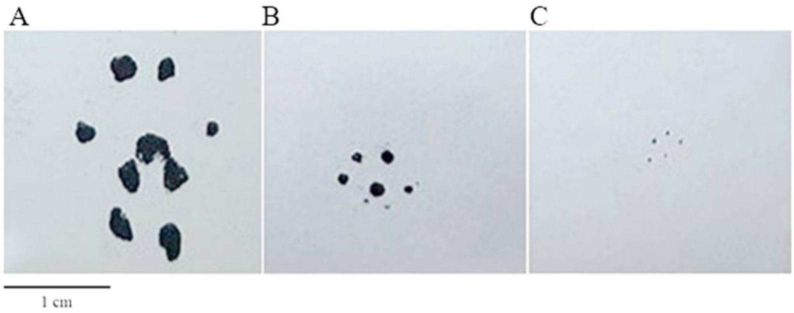

Figure S1A – Example of forefeet tracks of the three size-based groups: A - Large rodents, B - Medium rodents, C - Small rodents.

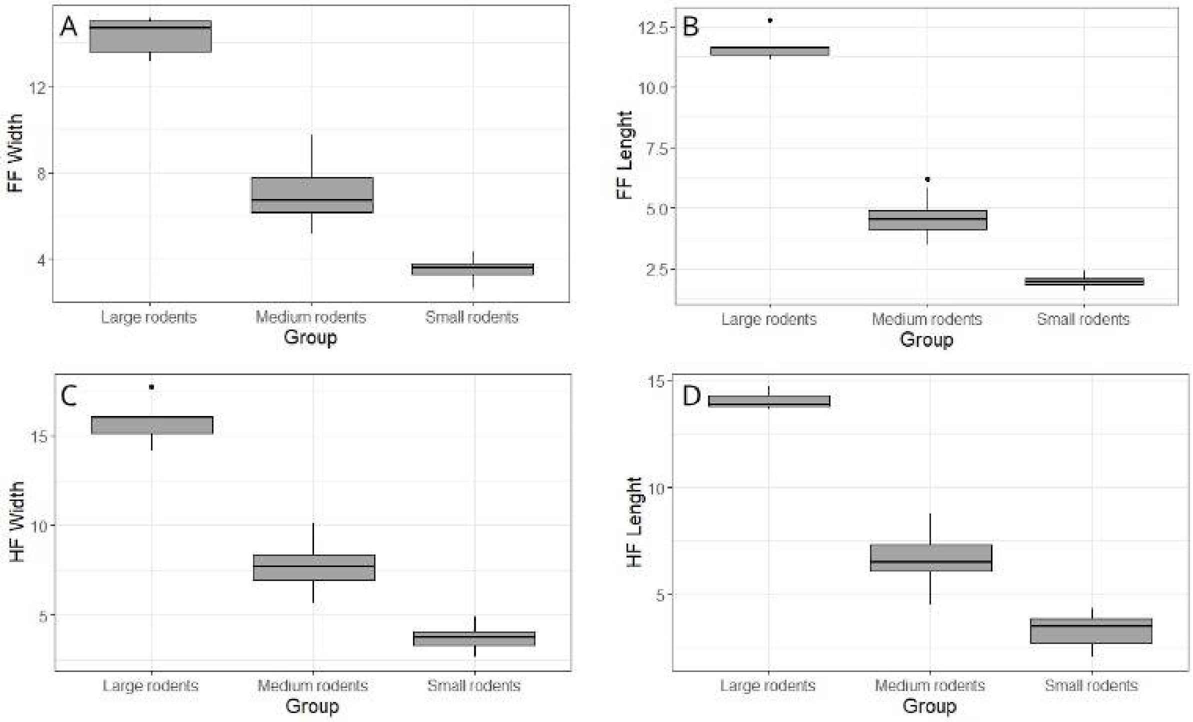

Figure S2A - Track measures in mm from the three size-based groups: A – forefoot (FF) width; B – forefoot length, C –hindfoot (HF) width; D – hindfoot length.

Table S1A - Dunn's test for the four track measurements between groups, with the corresponding test significance (*p*—value).

**FF Length**	Large Rodents		Medium Rodents	
	Test	*p*-value	Test	*p*-value
Medium Rodents	2.141881	0.0483		
Small Rodents	5.605665	<0.001*	8.241776	<0.001*
**FF Witdh**	Large Rodents		Medium Rodents	
	Test	*p*-value	Test	*p*-value
Medium Rodents	2.141839	0.0483		
Small Rodents	5.605555	<0.001*	8.241615	<0.001*
**HF Length**	Large Rodents		Medium Rodents	
	Test	*p*-value	Test	*p*-value

Medium Rodents	2.141902	0.0483		
Small Rodents	5.60572	<0.001*	8.241857	<0.001*

**HF Width**	Large Rodents		Medium Rodents	
	Test	*p*-value	Test	*p*-value
Medium Rodents	2.141812	0.0483		
Small Rodents	5.605486	<0.001*	8.241514	<0.001*

Table S2A - List of species occurring or possibly occurring in the region and their mean head to body length in mm (HB), hindfoot length in mm (HF) and weight in g (WT), with the two most abundant underlined [88]. Species names with a different color represent the species that were captured during the live-trapping sessions.

**Size-based group**	**Species**	**Common name**	**HB**	**HF**	**WT**
**Small rodents**	*Mus minutoides*	African pygmy mouse	54.4	12.5	6.2
	Dendromus mystacalis	Chestnut climbing mouse	57.5	17.1	8
	Dendromus melanotis	Grey climbing mouse	69	17.5	7.2
	Dendromus mesomelas	Brants' climbing mouse	75	20	11.3
	Steatomys pratensis	Fat mouse	94	16	32.9
**Medium rodents**	*Mastomys natalensis*	Natal multimammate mouse	107	22	36.4
	Grammomys dolichurus	Woodland mouse	113	24	32.9
	Aethomys namaquensis	Namaqua rock mouse	113	26	48
	Saccostomus campestris	Pouched mouse	114	21	48.5
	Lemniscomys rosalia	Single-striped grass mouse	126.3	26	54.2
	Gerbilliscus leucogaster	Bushveld gerbil	128.6	33.5	69.8
	Gerbilliscus brantsii	Highveld gerbil	134.6	35	79.9
	Thallomys paedulcus.	Sundevall's acacia rat	140.3	25.3	72.3
	Aethomys ineptus	Tete veld rat	147	30	83
**Large rodents**	*Mystromys albicaudatus*	White-tailed mouse	153.5	26.5	76.5
	Otomys irroratus	Southern African vlei rat	161	32	144
	Dasymys incomtus.	Common shaggy rat	165	33	158
	Rattus rattus	Black rat	165.3	31.7	132
	Petrodromus tetradactylus	Four-toed elephant shrew	192.9	54.8	198.3

3.1.1. Live-trapping

A total of 844 rodents were captured during the study. The highest number of rodents were captured in mixed farms (N= 408), followed by Mun-ya-wana game reserve (N= 340) and then communal lands (N= 96). In terms of size-based groups, medium sized rodents (N= 773) were dominated by *Mastomys natalensis* Smith, 1834 (N= 738), while small rodents (N= 71) were dominated by *Mus minutoides* Smith, 1834 (N= 68).

3.1.2. Comparison between methods (ink-tracking tunnels and live-trapping)

Considering the low trapping rate of small and large rodents, it was only possible to calculate the relative abundance for medium rodents. There is a significant positive correlation (r2 = 0.679, n = 18, $p < 0.001$) between the tracking index derived from the ink tunnel data and the relative abundance estimated from the trapping data, for medium rodents [89], which validates the use of the tacking index as a surrogate of rodents' abundance. Therefore, hereafter we will use abundance when referring to the tacking index. Furthermore, we detected the presence of small and medium size rodents in sampling plots were live-trapping did not, which reinforces this method efficiency.

PART B – Figures and tables additional to the manuscript

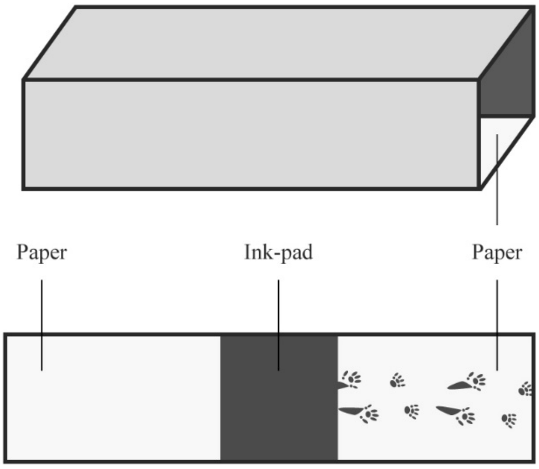

Figure S1B – Ink tracking tunnel scheme. Above is the ink tunnel seen from the outside, and at the bottom, the adhesive paper with the glue side up, the ink pad in the middle and some rodent footprints.

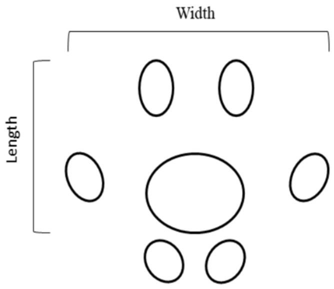

Figure S2B – Scheme of the method used to measure the 100 random tracks.

Table S1B – Species included in the wild ungulates category detected during the camera-trapping campaigns. Resulting variable was used as candidate variable in the modelling procedure.

Species	Weight	Supporting references
Nyala (*Tragelaphus angasii*),<br>Warthog (*Phacochoerus africanus*),<br>Impala (*Aepyceros melampus*),<br>Bushbig (*Potamochoerus larvatus*),<br>Common Redbuck (*Redunca redunca*),<br>Zebra (*Equus quagga*),<br>Wildebeest (*Connochaetes taurinus*),<br>Great Kudu (*Tragelaphus strepsiceros*),<br>Buffalo (*Syncerus caffer*),<br>Waterbuck (*Kobus ellipsiprymnus*)	45-600kg	[9,24,26]

Table S2B – Percentage of rodent detection in each type of area per group, based on the total number of ink tunnels installed (Mun-ya-wana game reserve = 98; Mixed farms = 50; Communal lands = 44).

Detection	Mun-ya-wana game reserve	Mixed farms	Communal lands	Total
**Medium**	69/98 = **70%**	42/50 = **84%**	34/44 = **77%**	145
**Small**	91/98 = **93%**	43/50 = **86%**	29/44 = **66%**	163

Table S3B – Linear regression models between size-based groups and area, and between groups per area. Significant values are in bold.

Model	Variable	Estimate	Std. Error	Z-value	*p*-value
**Small ~ Area**	(Intercept)	-0.818	0.307	-2.667	**0.008**
	Mun-ya-wana	0.888	0.368	2.415	**0.016**
	Communal lands	-0.212	0.460	-0.460	0.645
**Medium ~ Area**	(Intercept)	0.076	0.283	0.267	0.789
	Mun-ya-wana	-0.345	0.349	-0.989	0.323
	Communal lands	-0.662	0.423	-1.565	0.118
**Small ~ Medium (Mixed farms)**	(Intercept)	-1.526	0.602	-2.536	**0.011**
	Medium	1.281	0.875	1.463	0.143
**Small ~ Medium (Mun-ya-yana)**	(Intercept)	-0.214	0.307	-0.696	0.487
	Medium	0.656	0.537	1.223	0.221
**Small ~ Medium (Communal Lands)**	(Intercept)	-0.920	0.536	-1.718	0.086
	Medium	-0.312	1.195	-0.261	0.794
